# Evaluation of Emotional Intelligence and Job Satisfaction in Employees of Kashan Hospitals

**DOI:** 10.17795/nmsjournal11977

**Published:** 2014-04-17

**Authors:** Fatemeh Sadat Ghoreishi, Ali Reza Zahirrodine, Fatemeh Assarian, Seyed Gholam Abbas Moosavi, Maryam Zare Zadeh Mehrizi

**Affiliations:** 1 Department of Psychiatry, Kashan University of Medical Sciences, Kashan, IR Iran; 2 Department of Psychiatry, Shahid Beheshti University of Medical Sciences, Tehran, IR Iran; 3 Department of Biostatistics and Public Health, Kashan University of Medical Sciences, Kashan, IR Iran; 4 Department of Psychiatry, Kashan University of Medical Sciences, Kashan, IR Iran

**Keywords:** Job Satisfaction, Emotional Intelligence, Hospital

## Abstract

**Background:**

Job satisfaction and emotional intelligence are two important variables in organizational behavioral studies, and are key factors in promoting the efficiency of organizations.

**Objectives:**

The present study was conducted in order to determine the job satisfaction and emotional intelligence of employees of Kashan hospitals in 2011.

**Materials and Methods:**

This cross-sectional study was performed on 121 employees of Kashan hospitals who were selected using random stratified method. In this study, Bar-on emotional intelligence and job satisfaction questionnaires were used. The data were analyzed using statistical methods such as odds ratio, Chi-square and Fisher's exact test.

**Results:**

The majority of employees (76%) had moderate emotional intelligence while 88.2% of them had moderate job satisfaction. In this study, there were no significant relations between emotional intelligence and variables such as sex, education, and marital and job status (P > 0.05) but significant relations were found between the age and emotional intelligence (P = 0.01). Furthermore, there was no significant relation between job satisfaction and demographic variables. Moreover, no significant relation was found between the emotional intelligence and job satisfaction (P > 0.05).

**Conclusions:**

As the majority of the staff had average level of job satisfaction and emotional intelligence and others were lower than average, it seems necessary for authorities to explore the reasons for job dissatisfaction to prevent job burnout, depression and developing a sense of helplessness in the staff. It is also recommended to hold educational workshops for the staff especially who are younger than 40 years to promote their emotional intelligence.

## 1. Background

Emotional intelligence (EI) is a new concept, which has been used and defined in the management literature since 1990. Different studies have demonstrated that emotional intelligence is one of the virtues associated with success in life. Developing emotional intelligence among the staff can solve many problems in health, education, and management. There is a growing body of evidence regarding the emotional aspects of work in an organization. Although, few management researchers have accepted this concept, the concept of emotional intelligence has been used by the administrative authorities in many workplaces to explain issues related to the job satisfaction, performance, absenteeism, organizational commitment and leadership (1, 2). In the context of the emerging ‘affective revolution’ in social and organizational psychology, emotional intelligence is proposed as an important predictor of key organizational outcomes including job satisfaction (3-6). Emotional intelligence is considered to play a significant role in the work environment. It is a basic requirement in any profession that is based on human relations especially in the nursing. Emotions play an important role in the nursing profession which requires both technical expertise and psychologically oriented care, so an emotionally intelligent nurse is a person who can work in harmony with his/her thoughts and feelings (7). Emotional intelligence is the ability to perceive, evaluate and express emotions rapidly and to understand and manage them using emotional information and to direct thoughts, actions and affects to have a successful work experience. There are two types of EI measure: ability and trait. The trait EI theory distinguishes between the intrapersonal and interpersonal domains. Intrapersonally, use of emotions can lead to regulating stress and negative emotions so that one can perform better at work. Interpersonally, the ability to understand and respond appropriately to the motivations and feelings of other people can lead to the appraisal and regulation of emotions in others and achieving maximum performance (8, 9). Researchers specifically propose that such an ability can predict work outcomes, such as intention to quit, the staff turnover, job satisfaction and job performance (10, 11). Studies have shown that the selection of personnel based on the emotional intelligence had better results compared to the traditional methods which may have a greater reliance on cognitive abilities and technical knowledge, while this issue has been rarely studied in the nursing profession (7, 12).

On the other hand, one of the important issues in the field of organizational success is job satisfaction. Job satisfaction can be defined as the extent to which employees like their jobs. It is an emotional state of individuals that is enhanced by achieving favorable results at work and the feelings of belonging to a functioning work place (13).Traditional job satisfaction pointed to the feelings of an individual towards his/her job. Two sets of factors are considered to influence job satisfaction: intrinsic (recognition, tasks and responsibility) and extrinsic factors (working conditions, company policies and salary) (14). Internal job satisfaction is an internal desire to perform a task which deals with pleasure and is related to internal motivation. External factors are defined as those external benefits provided to the professional staff by the organization. These factors are unrelated to the task and include money, good scores and other rewards. Hospitals that have higher job satisfaction scores among the employees, have a better quality of care and more favorable outcomes (15). Studies have shown that high degree of emotional exhaustion can predict lower self-rated performance and higher intention to quit work which is a consequence of low job satisfaction (16). Thiebaut has demonstrated in his studies that scores in emotional intelligence surveys can predict 36% of organizational outcome factors, 25% of the quality of interpersonal relationships, 34% of salaries and rewards and 42% of job satisfaction (17). Sy et al. in a study conducted on the staff from various companies, showed that people with high emotional intelligence have more vitality, joy and independence at work, have better job performance and will be more stress resistant (10). Findings of the study of Lopes et al. supported the link between emotional intelligence and job satisfaction in a group of managers (18). Morever, Guleryuz found that job satisfaction is a mediator between regulation of emotion and organizational commitment (19). Ignat showed that teachers with a good level of emotional intelligence had a positive attitude toward work and had more job satisfaction (20). Syed Hassan also showed that four dimensions of emotional intelligence significantly influence work values among educators (21). However, other studies in this field done by Aghdasi (22) and Casper (23) did not find a significant relation between job satisfaction and emotional intelligence. There are several lines of evidence supporting that emotional intelligence is necessary for success in many professions and achieving higher job satisfaction. Job satisfaction of the employees affects the efficiency of the organizations which can enhance the level of organizational success, individual efficiency, employees’ commitment to the organization and ability to learn occupational skills.

## 2. Objectives

The current study was aimed to evaluate the degree of emotional intelligence and job satisfaction in the staff of Kashan hospitals. 

## 3. Materials and Methods

This was a cross-sectional study carried out on the staff of three general hospitals in Kashan (Shahid Beheshti, Akhavan and Mathini) during 2010-2011. The sample size was calculated to be 121 subjects using the parameters as follows: z = 1.96, P = 0.82, q = 0.18, d = 0.05. Subjects were selected by stratified random sampling and randomized number tables. Inclusion criteria were as follows: all the administrative and non-administrative staff except for the physicians (because of different educational and income levels). Subjects were classified into administrative and non-administrative staff categories; administrative staff included employees who provide support services and were not directly involved in the health care services. Non administrative staff included individuals working in the section of health services, such as nurses. We had separate lists of subjects from the administrative and non-administrative sections of each hospital. The proportion of staff from each hospital was determined via Equation 1. 

Equation 1. ni = ki/N × n

Data were collected using a questionnaire consisting of three parts:

1. Demographic information including the age, sex, education, occupation, marital status, employment history, previous job status, physical-psychological health, economic status, housing status.
2. Job satisfaction questionnaire consisted of 28 three- choice and 2 eleven-choice questions. Each three choice question was scored according to the order of the selected choice such as 1, 3, or 5, while questions 12 and 13 were scored as 0, 5, 10, 15, 20, 25, 30, 35, 40, 45, or 50. A total score of 51-84 was regarded as being low, 85-144 moderate, 145-175 high, and more than 175 as abnormal job satisfaction. The validity of this questionnaire was verified by professionals and test-retest method was used to determine its reliability. In this regard, 40 questionnaires were distributed initially among the study subjects, and once again after a week to the same sample population, and Pearson correlation was calculated to be 0.95 (P > 0.001).
3. Emotional Intelligence questionnaire had 133 questions and was the first cross-culturally validated questionnaire to assess emotional intelligence. There were five choices available for each question (never, rarely, sometimes, often and always) and the scoring scale was based on the Likert method, where each question was scored between 1-5, either in order of the choices or vice versa. This questionnaire was designed in 1997 by Bar which gives a total score for emotional intelligence and has five components and 15 sub-scales.

The five components of the questionnaire contain 15 subscales such as intrapersonal relation scale (emotional self-awareness, self-esteem, assertiveness, independence and prosperity), interpersonal relations scale (empathy, social responsibility, and interpersonal relationships), compatibility scale (true adaptability, flexibility, and problem solving), cope with pressure scale (stress tolerance and impulse control) and general mood scale (happiness, optimism). The questionnaire’s reliability and validity coefficients have been previously obtained using different methods (24). In the study of Samari and colleagues conducted using this questionnaire in 2010, the Cronbach's alpha was calculated as 0.93% (25). A copy of the questionnaire was distributed among the participants, and after explaining the purpose of the study to them and obtaining an informed consent, they were asked to complete the questionnaire. They filled out questionnaires in 30 minutes.

### 3.1. Ethical Considerations

The study protocol was approved by the Ethics Committee of Kashan University of Medical Sciences. All ethical issues such as obtaining informed consent and avoiding plagiarism were followed. The respondents were anonymous and all the information were kept confidential in this study. All participants signed a written informed consent befor participation in the study.

### 3.2. Data Analysis

Data were analyzed using SPSS-16 software and statistical analysis such as odds ratio, Chi square and Fisher Exact test.

## 4. Results

One hundred twenty one subjects participated in this study with mean age of 32.03± 7.02. Table 1 provides the distribution of demographic variables in these subjects. The emotional intelligence of the majority of staff (76%) was moderate regarding the emotional intelligence subscales; 50% had moderate emotional intelligence in mood scale; 68% had low emotional intelligence (lower than the average) in coping with stress scale; 68% had low emotional intelligence (lower than the average) in consistency scale; and 57% had moderate emotional intelligence in interpersonal scale; and in the intrapersonal scale 80% had lower than the average level of emotional intelligence.

Sixty-eight women (75.5%) and 25 men (81%) had moderate emotional intelligence, the frequency of which was higher in men compared to women, but this difference was not statistically significant (P = 0.56). The prevalence of moderate emotional intelligence in non-administrative staff (80.5%) was more than the administrative staff (70.5%) which was not statistically significant (P = 0.20). In this study, there were no significant relations between emotional intelligence and variables such as sex, education and marital and job status (P > 0.05) but significant relations were found between the age and emotional intelligence (P = 0.01). The majority of participants (n = 107) had moderate job satisfaction (88.4%) and 14 staff (11.6%) had low job satisfaction.

**Table 1.** Frequency Distribution of Demographic Variables of the Study Population

Demographic Variables	No. (%)
**Gender**	
Male	90 (74%)
Female	31 (26%)
**Age, y**	
≤ 40	102 (84%)
> 40	19 (16%)
**Education status**	
Under graduate	18 (15%)
Post graduate	103 (85%)
**Job status**	
Administrative	44 (47%)
Non-administrative	77 (63%)
**Marital status**	
Single	33 (28%)
Married	88 (72%)
**Occupational background, y**	
≤ 10	91 (75%)
> 10	30 (25%)
**Previous jobs status**	
Yes	19 (16%)
No	102 (84%)
**Physical health status**	
Healthy	107 (88%)
Unhealthy	14 (12%)
**Economic status**	
Income ≥ costs	81 (66%)
Income < costs	40 (34%)
**Housing status**	
Owner	93 (76%)
Non-owner	28 (24%)

In this study there were no significant relations between age, sex, education and job status with job satisfaction, but there was a significant relation between the marital status and job satisfaction (Table 2). In the evaluation of relation of the job satisfaction with emotional intelligence and its sub scales the following results were obtained:

As showed in Table 3, a significant relation was not found between job satisfaction and emotional intelligence and its subscales (P > 0.05).

**Table 2.** Frequency of Job Satisfaction among Employees Based on the Related Factors in Kashan Hospitals in 2011 ^a^

Job Satisfaction	Low, No. (%)	Moderate, No. (%)	P Value	OR	CI
**Gender ^b^**			1	0.76	0.2-0.29
Male	11 (12.2)	79 (87.8)			
Female	3 (9.7)	28 (90.3)			
**Age, y ** ^**b**^			0.46	1.55	0.38-6.18
< 40	11 (10.8)	91 (89.2)			
> 40	3 (15.8)	16 (84.2)			
**Education status ** ^**b**^			0.436	0.59	0.14-2.39
Graduate	3 (16.7)	15 (83.3)			
Post graduate	11 (10.7)	92 (89.3)			
**Job status ** ^**c**^			0.06	3.8	0.82-18.2
Administrative	2 (4.5)	42 (95.5)			
Non-administrative	12 (15.6)	65 (84.4)			
**Marital status ** ^**b**^			0.109	5.54	0.69-44.2
Single	1 (3)	32 (97)			
Married	13 (14.8)	75 (85.2)			

^a^ Abbreviations: CI, Confidence Interval; OR, Odds Ratio.

^b^ Fisher exact test.

^c^ Chi-square test.

**Table 3.** Relation of Job Satisfaction with Components of Emotional Intelligence Scale in Hospital Employees

Job Satisfaction	Low, No. (%)	Moderate, No. (%)	P Value	OR	CI
**General emotional intelligence ^a^**			0.18	4.3	0.58-35.1
Low	1 (7.1)	13 (92.9)			
Moderate	27 (25.2)	80 (74.8)			
**General mood ^b^**			0.54	1.4	0.45-4.34
Low	6 (9.8)	55 (90.2)			
Moderate	8 (13.3)	52 (86.7)			
**Coping with pressure ^a^**			0.76	1.2	0.38-4
Low	9 (10.8)	74 (89.2)			
Moderate	5 (13.2)	33 (86.8)			
**Compatibility ^a^**			0.37	1.7	0.54-5.3
Low	8 (9.6)	75 (90.4)			
Moderate	6 (15.4)	33 (84.6)			
**Interpersonal relationships ^b^**			0.28	0.51	0.155-1.73
Low	7 (13.5)	45 (86.5)			
Moderate	5 (7.5)	62 (92.5)			
**Intrapersonal relationships ^a^**			1	1.1	0.28-4.36
Low	11 (11.3)	86 (88.7)			
Moderate	3 (12.5)	21 (87.5)			

^a^ Fisher exact test.

^b^ Chi-square.

## 5. Discussion

This study was conducted in Kashan hospitals in order to evaluate the level of emotional intelligence and job satisfaction. The emotional intelligence of the majority of staff was at average level and that of other staff was low. In evaluation of emotional intelligence subscales in staff with average level of emotional intelligence, the highest scores were obtained in interpersonal relationships (57%), general mood (50%) and other aspects of emotional intelligence were less than average. In this study, there was significant relation between emotional intelligence and age, so the staff over 40 years of age had higher emotional intelligence than those under 40 years. In a study conducted by Casper in 2007, no relation between emotional intelligence and demographic variables such as age was found, which is in contrast to our results (23). A higher level of emotional intelligence in persons older than 40 years can be likely attributed to the fact that as one becomes older he gains more experience in interpersonal relationships and a better ability to deal and cope with stress, and hence a higher level of emotional intelligence. Also this survey showed that emotional intelligence in people with medical career (nurses) was more than administrative staff but the difference was not statistically significant. Some studies have shown that emotional intelligence helps nurses to develop therapeutic relationships with patients and their families and to manage stress (7). Ranjbar Ezzatabadi showed that the emotionally intelligent nursing staff are more likely to deliver high quality services and emotional intelligence has direct effect on the quality of the delivered services. Meanwhile job satisfaction and communication skills had an intermediate level contribution to the level of emotional intelligence and service quality (1). Another important finding of this study was job satisfaction of the staff, which, in 88.25% of the staff was reported to be at average level and others were reported to be at low level. In a study conducted by Lorber there was a medium level job satisfaction in the administrative staff and nursing employees which is similar to the results of our study (26). The results of this study showed that the majority of staff reported moderate job satisfaction, while Krogstad study in Norway showed a high level of job satisfaction among hospital staff, which is not consistent with the results of our study (27).

There were no significant relations between job satisfaction and demographic variables. In this regard, Hodson study in 1989 is consistent with the results of our study (28). Schiestel also did not find any significant relation between job satisfaction and gender, annual income and employment status (29). However, Keshani study in 1998 indicated that there is significant relation between job satisfaction and gender, which is not consistent with our findings (30). On the other hand, there was no significant relation between job satisfaction and emotional intelligence and its subscales. The results of our study are consistent with the results of several studies in this field, such as Aghdasi (22) and Casper (23). Furthermore Masroor Alam demonstrated that there is no significant relation between intrapersonal factors, and stress management with job satisfaction, which is consistent with our results. The above researcher concluded that emotional intelligence can predict 16% of the variance of job satisfaction by itself and only the general mood subscale, among five subscales of emotional intelligence, can predict job satisfaction (31). However, several studies showed that there is a significant relation between job satisfaction and emotional intelligence, which was not consistent with the results of our study (10, 19, 32-36). Trivellas found that there is a positive relation between only two dimensions of emotional intelligence and job satisfaction (11). Cekmecelioglu also showed a significant positive relation between emotional recognition –the first dimension of emotional intelligence and internal job satisfaction but he did not found any relation between emotional intelligence and external job satisfaction (37). As such, it seems that the results of the studies which have examined relation between emotional intelligence and job satisfaction are different. A number of studies have observed weak to modest relation between EI trait measures and job satisfaction (5, 8, 38). Perhaps one reason for the difference in the results is the use of different tools to measure emotional intelligence and job satisfaction. Furthermore, it could be likely due to the difference in the studied populations, work places, rules and conditions of organizations. It appears that, in addition to emotional intelligence and values of the staff, job satisfaction is also influenced by culture and structural status of organizations. Perhaps, the reason for the inconsistency among the findings of different studies on the association between emotional intelligence and job satisfaction is the fact that it is affected by various factors. In Iran, the effect of emotional intelligence on job satisfaction is of lesser degree resulting from lower income, harder working conditions, and fewer facilities. On the other hand, organizational determinants like management approach, mode of supervision, job autonomy, and task delegation are also among the factors influencing job satisfaction. Overall, since the majority of the staff had an average level of job satisfaction and emotional intelligence and others were lower than average, it seems necessary for authorities to discern the factors affecting job dissatisfaction to prevent job burnout, depression and feeling of helplessness in the staff. It is also recommended to hold educational workshops for the staff especially who are younger than 40 years old to promote their emotional intelligence.
